# Microstructure and Texture Evolution of X85MnAl29-9 Steel During Aging

**DOI:** 10.3390/ma17225646

**Published:** 2024-11-19

**Authors:** Małgorzata Witkowska, Kinga Chronowska-Przywara, Joanna Kowalska, Anna Zielińska-Lipiec

**Affiliations:** 1 Faculty of Metals Engineering and Industrial Computer Science, AGH University of Krakow, Av. Mickiewicza 30, 30-059 Krakow, Poland; joannak@agh.edu.pl (J.K.); alipiec@agh.edu.pl (A.Z.-L.); 2 Faculty of Mechanical Engineering and Robotics, AGH University of Krakow, Av. Mickiewicza 30, 30-059 Krakow, Poland

**Keywords:** manganese steel, spinodal decomposition, nano-carbides, microstructure, texture

## Abstract

The research presented in this paper is part of a larger project concerning high-manganese alloys with different chemical compositions (mainly in manganese content from 21 to 31 wt.%). The presented examination results concern the analysis of the microstructure and textures in high-manganese X85MnAl29-9 steel, an age-hardenable steel, during aging at 550 °C for various times. X85MnAl29-9 steel was first hot rolled and subsequently cold rolled up to a 30% reduction. The samples were aged after deformation at 550 °C for various times in an argon atmosphere and cooled in air. The studies include X-ray phase analysis, texture measurement and observation of the microstructure by light microscopy, transmission electron microscopy (TEM) and scanning electron microscopy (SEM), as well as microhardness measurement. Research using scanning and transmission electron microscopy identified carbides in the analyzed samples. The results indicate that, when aging takes place, precipitation of κ′-carbide in an austenitic matrix and carbide κ at grain boundaries occurs. The appearance of satellites on diffraction patterns suggests that (Fe, Mn)_3_AlC nano-carbides are formed within the austenite matrix by a spinodal decomposition mechanism after the alloy is subjected to long-term aging, which is a key element for structure analysis in the design of safety systems. The use of shorter aging times (up to 24 h) leads to an increase in hardness caused by the precipitation of small κ′-carbide particles in the matrix. However, long aging times (100 h) lead to an increase in the precipitation of the carbide phase (κ and κ′), i.e., the steel becomes overage, which results in a decrease in hardness.

## 1. Introduction

The automotive industry is a dynamically developing branch of the global economy. The last 30 years have been characterized by, first and foremost, an increasing level of passenger safety and newer elements of accessories. This has resulted in an approximately 30% increase in vehicle mass, which has contributed to increased fuel consumption and, ipso facto, environmental damage. Therefore, the increase in demand for light, high-strength construction materials has contributed to the development and production of advanced construction materials without an increase in financial outlays. The new produced materials fulfill requirements related to reductions in vehicle mass and maintaining the level of strength properties without causing changes in cost. All these conditions are fulfilled by high-manganese austenitic steels of the following types: TRIP, TWIP and TRIPLEX [1,2,3,4,5,6,7]. Depending on the content of Mn, Al and C and the manufacturing process, Fe-Mn-Al-C steels can be ferritic or austenitic with duplex or triplex phases [8,9,10,11,12,13,14,15,16]. The addition of Al reduces the mass densities of the steels, promotes ferrite formation and increases stacking fault energy (SFE) in austenite [17,18,19]. The high alloy content of C and Al promotes the precipitation of coherent nanoscale (Fe, Mn)_3_AlC carbides within the austenite during aging treatment. It is known that Mn and C are strong austenite formers, and C can also increase the SFE [20,21,22]. High-manganese steels are promising in terms of their applications whenever, apart from high material strength, high plasticity is also required. These alloys possess specific energy absorption values of about 0.43 J/mm^3^. High-manganese steels can be used in parts critical for safety by ensuring the high absorption of energy by structural parts whose task is to absorb energy during abrupt deformation [1,23].

TRIPLEX steels, containing 0.7–2% C, 18–35% Mn and 8.5–12% Al, constitute a group of steels with a high level of strength properties in the order of between 700 and 1100 MPa, and good plastic properties (60%) [3,24,25]. Furthermore, one of their features is a low density level (6.5–7 g/cm^3^). The microstructure of TRIPLEX steels is constituted by an austenitic matrix, containing a small quantity of ferrite Fe (Al, Mn) and a fine dispersion of nano-carbide κ (Fe, Mn)_3_AlC. Under some compositions, temperatures, heating and/or cooling processes these phases may coexist. Carbide is an L’1_2_-ordered phase with Al atoms located at the corners, Fe and/or Mn atoms located at the face centers and C atoms located at the body center in the lattice. κ-carbide precipitates have a cube-on-cube orientation relationship with the γ-matrix, because both phases have a cubic lattice with similar lattice parameters [3,26,27,28]. The strengthening effect is achieved as a result of a coherency strain field developing between the γ-matrix and κ-carbide, which impedes dislocation glide. In order to enhance their strength properties, these steels are subjected to aging within the following scope of temperatures: 500–750 °C [29,30,31]. The results of this thermal treatment are small-dispersive precipitates of carbide κ′ arranged in the austenitic matrix.

Many researchers have believed that the formation of intra-granular κ′-carbide is through spinodal decomposition and the following ordering reaction [27,32,33]. A spinodal reaction causes the modulation of C and Al within the austenite [26,27,34], which decomposes the high-temperature austenite into two low-temperature FCC austenite phases: the solute-lean (C and/or Al) phase γ′ and the solute-rich phase γ″. Then, the solute-rich phase γ″ transforms into the L’1_2_-ordered phase, in which Al and Fe/Mn atoms are located at corner and face center sites, respectively. Finally, the κ′-carbide forms by the further ordering of C atoms. The spinodal distribution occurs uniformly throughout the material, creating a modulated structure [26,35]. Due to the precipitation of (Fe, Mn)_3_AlC carbides within the austenite matrix, the strength of the alloy is considerably increased without a significant loss in ductility [20,26,27]. The factors exerting a significant influence upon the precipitate of nano-carbides are chemical composition (aluminum and carbon content), temperature and the time of aging [11,36]. Frommeyer et al. [3] examined the influence of chemical composition on the properties of Fe-Mn-Al-C steels with different percentages of Mn (18–28%), Al (9–12%) and C (0.7–1.2%). They found that the good strength properties of 700–1100 MPa are caused by the presence of nano-carbides in the steel structure. Li et al. [37] examined the strength properties of Fe-27Mn-10Al-1C steel for various aging temperatures. They found that an increase in the process temperature led to an increase in κ′- and κ-phase precipitation, which resulted in an increase in strength properties during tensile deformation at the expense of plastic properties. Similarly to Li et al. [37], other authors [38] also stated that with the extension of the aging time, carbides precipitate not only coherently within the austenite matrix (κ′), but also along grain boundaries (κ). Chang et al. [24] studied the effect of heat treatment on the mechanical properties of steel with the composition Fe-9Al-28Mn-1.8C. Temperature aging at 450 °C at shorter times led to an increase in strength properties; however, extending the time beyond 15 h led to a decrease in strength properties (UTS, YS), which was caused by the precipitation of κ-carbides along the austenite grain boundary. Fine κ′-carbides increase strength; however, thick κ-carbides distributed along the grain boundaries lead to its decrease [3]. The intergranular κ-carbides are much coarser and can result in crack initiation and propagation. This was confirmed by research conducted on Fe-20Mn-11.5Al-1.2C steel by Kim et al. [25] regarding the correlation between microstructure and properties. Similarly, phases such as DO_3_ and β-Mn, which lead to degradation of the mechanical properties, could precipitate depending on the chemical composition or process conditions during heat treatment. Depending on their shape, location and size, they can have different effects as they can induce strengthening as well as embrittlement [11,16,36,37]. The studies carried out indicate changes occurring in the material after aging, which is important when selecting heat treatment conditions for individual industrial applications, especially with regard to passive safety systems in vehicles.

The objective of this investigation was to analyze changes in the microstructure and texture taking place in the course of aging X85MnAl29-9 high-manganese steel. Changes in the microstructure of aged steel were analyzed using light (LM), scanning (SEM) and transmission electron microscopy (TEM), as well as diffraction tests. The influence of the obtained microstructure on the properties of steel was also analyzed by microhardness measurements. Additionally, changes in the main components of the crystallographic texture of austenite with increasing aging times were determined.

## 2. Materials and Methods

The research material was a high-manganese steel melted in a laboratory vacuum induction furnace. The chemical composition of the steel is shown in Table 1. The chemical analysis was performed by ICP (Inductively Coupled Plasma) optical emission spectroscopy using an Ultima 2 Jobin Yvon spectrometer (HORIBA Jobin Yvon Inc., Edison, NJ, USA). The chemical composition of the steel was designed based on an analysis of the available literature data [3,7,11,20,39,40,41], so as to obtain a purely austenitic structure in the initial state. Elements such as manganese and carbon are responsible for stabilizing austenite, while aluminum reduces the weight of the alloy. The addition of aluminum above 5% additionally promotes κ-carbide precipitation [41].

After melting, a cast ingot was annealed at 1150 °C for 7 h in an argon atmosphere. Next, it was hot rolled at a temperature of 1250 °C (50% of reduction) and cold rolled until a deformation of 30% was achieved. Following this, the samples were aged at 550 °C for various periods up to 100 h.

For the purpose of diffraction testing, a Siemens D500 diffractometer (Siemens, Munich, Germany) and monochromatic radiation generated by a tube with a copper anode (λ_Kα_ = 0.154 nm) were applied.

The texture measurements were conducted with the diffractometric Schulz reflection method on a Bruker D8 Advance diffractometer (Bruker AXS GmbH, Karlsruhe, Germany) equipped with a Euler circle and a tube equipped with a cobalt anode (λ_Kα_ = 0.179 nm). Incomplete pole figures were recorded as {111}, {200}, {220} and {311} for austenite, and, on their basis, the orientation distribution function (ODF) was calculated.

The microstructure observations were performed with a DM4000M light microscope (Leica Microsystems Wetzlar GmbH, Weltzlar, Germany), an FEI Versa 3D FEG scanning electron microscope (Waltham, MA, USA) and also with a JEM 200CX transmission electron microscope (JEOL, Tokyo, Japan). For optical metallographic observations, specimens were mechanically ground with 400 to 4000 SiC grit paper, followed by mechanical polishing using a 3 μm and 1 μm diamond suspension, and etched with a Nital solution (5% for the deformed sample and 10% for aging samples).

TEM samples were prepared by the mechanical grinding of the specimens to a thickness of 30 µm and electropolishing with a double electropolisher (Tenupol-5, Struers^TM^, Ballerup, Denmark) at 10 °C, using a solution containing CH_3_COOH (90 vol.%) and HClO_4_ (10 vol.%). Electron microscope investigations were performed on longitudinal sections.

Vickers microhardness measurements were performed using a load of 1 newton to elucidate the effect of aging heat treatment on mechanical properties. As well as these, microhardness measurements with an INNOVATEST microhardness tester (Innovatest Europe BV, Maastricht, the Netherlands) were performed. The microhardness (HV0.1) is the average from 10 measurements for deformed and aged alloy, with the standard deviation calculated for each sample.

## 3. Results and Discussion

### 3.1. X-Ray Investigation

**Figure 1:**
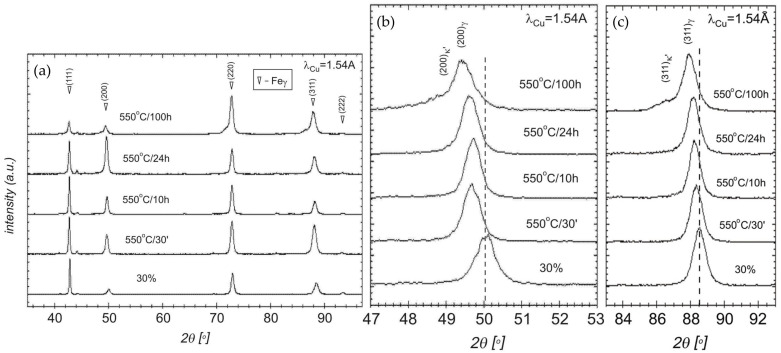
X-ray diffraction patterns of X85MnAl29-9 steel after (**a**) 30% reduction and aging at 550 °C for various times. (**b**,**c**) X-ray diffraction profiles around the (200)γ and (311)γ Bragg reflection after rolling deformation and aging at 550 °C, respectively.

The conducted X-ray phase analysis and the analysis of diffraction line profiles make it possible to assess changes in the phase composition depending on the time of aging (Figure 1). In the diffractogram, the strongest peak was (220)γ for the sample after a deformation of 30% (Figure 1a). Next to it, strong peaks of (311)γ and (111)γ were also observed. In the diffractograms made for the samples annealed at a temperature of 550 °C after different time periods, certain changes were observed in the intensity of particular peaks; namely, for samples annealed for 30 min (sample 550 °C/30′) and 10 h (sample 550 °C/10 h), the strongest peaks were still the following ones: (220)γ, (311)γ and (111)γ. For the sample annealed for 24 h (sample 550 °C/24 h), a strong peak was (200)γ, whereas for the sample after 100 h (sample 550 °C/100 h) it was a (220)γ peak. In Figure 1b,c, diffraction profiles within the angular scope 2θ from 47 up to 53° and 83° up to 93°, encompassing the occurrence of the (200)γ and (311)γ peaks, are presented. In the image of these peaks, the greatest changes taking place in the samples during the aging process are shown. With an increasing time of annealing up to 24 h, an increase in the peak intensity was observed, whereas, after 100 h of aging, its intensity decreased.

The peak of κ′-carbides, including (311), is detectable only in the sample after aging for 100 h, which is probably due to the smaller fraction of the ordered nano-sized κ′-carbides in the other samples. The presence of carbides in these samples can be proven by the broadening and shifting of austenitic peaks to smaller Bragg angles. This also proves the precipitation of κ′-carbide as a result of spinodal decomposition. The C atoms at the body center of κ′-carbide significantly increased the lattice parameter, providing slight peak shifts of the diffraction angle with the FCC γ-phase [42,43,44]. However, it must be noted that XRD measurements are not suitable to detect the small volume fractions of ordered nano-carbides that are present in this material after aging.

Initially, the intensity ratios I_γ111_/I_γ200_ and I_γ111_/I_γ220_ were calculated for the studied samples and compared with the indexes for a pattern. The occurring differences provided us with some preliminary information about the material being textured (Table 2). The greatest differences in the intensity ratios occurred for the extended times of aging, 24 and 100 h. 

### 3.2. Microstructure Analysis

**Figure 2:**
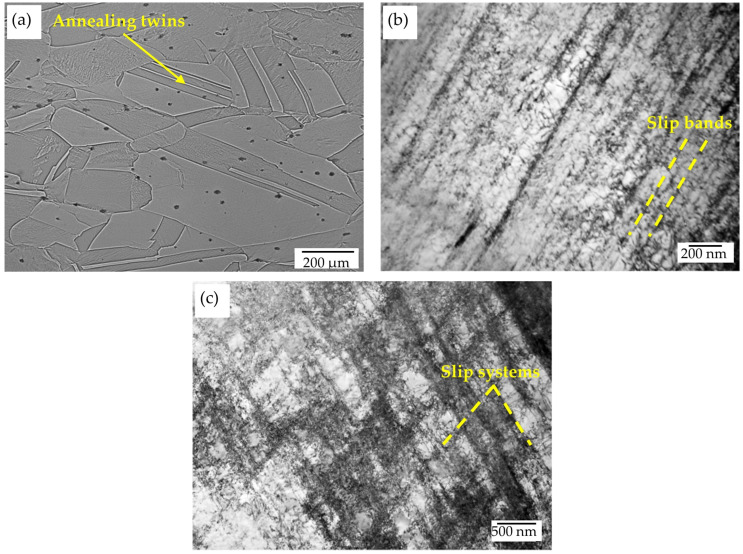
Microstructures of X85MnAl29-9 steel after 30% reduction: (**a**) micrograph obtained by means of LM and (**b**,**c**) TEM micrographs—BF.

**Figure 3:**
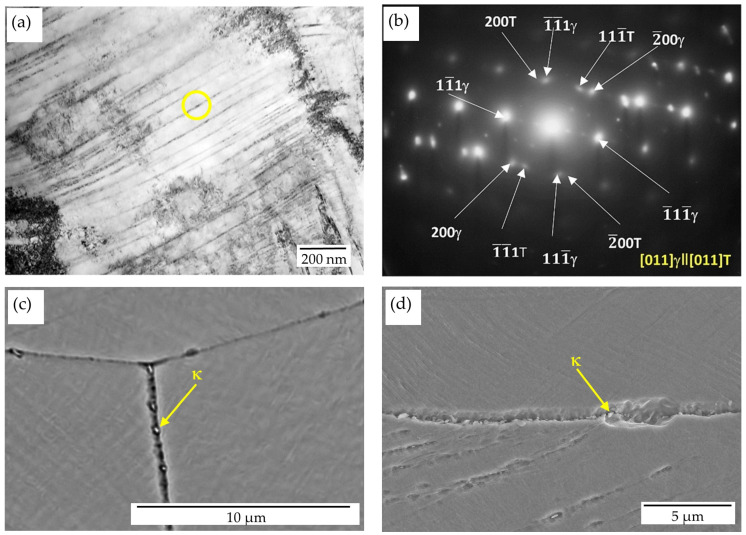
Microstructures of X85MnAl29-9 steel after 30% reduction and aging at 550 °C for 10 h: (**a**) TEM micrographs—BF, (**b**) SADPs taken from areas indicated by a circle, (**c**,**d**) SEM micrograph.

**Figure 4:**
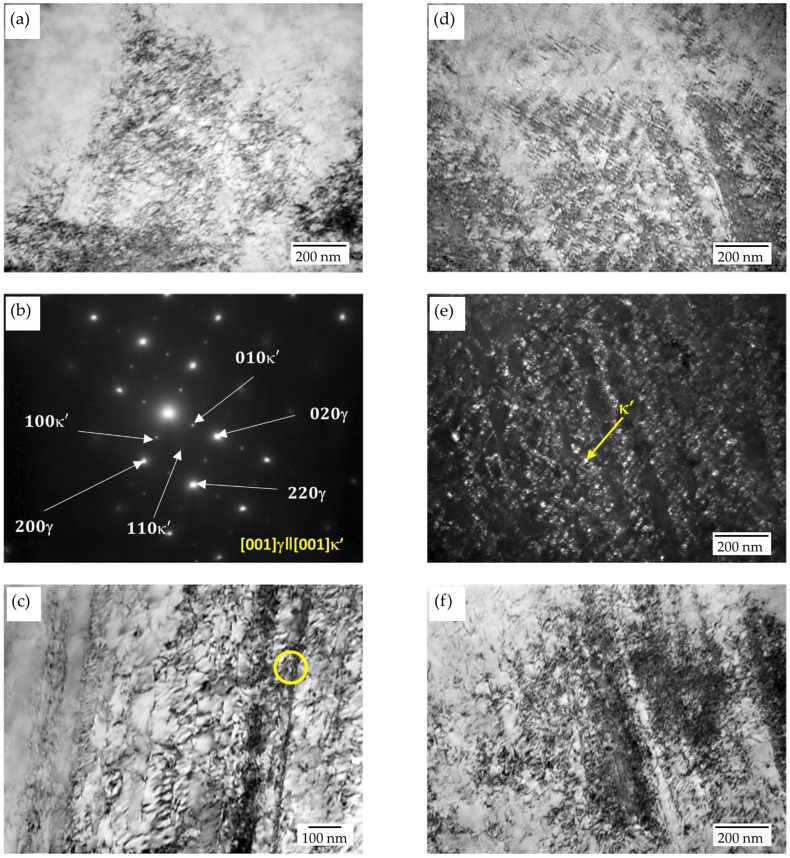
Microstructures of X85MnAl29-9 steel after 30% reduction and aging at 550 °C for 10 h: (**a**) TEM micrographs—BF, (**b**,**c**) SADPs taken from areas indicated by a circle and BF. And after aging at 550 °C for 100 h: (**d**) TEM micrographs—BF, (**e**,**f**) DF and BF.

The microstructures for the selected X85MnAl29-9 steel samples after deformation and then after aging are presented in Figure 2, Figure 3 and Figure 4 by light microscopy image (LM) and transmission electron (TEM) and scanning electron microscopy (SEM). 

After 30% of deformation, the material was characterized by flattened austenite grains, which were elongated in the rolling direction. In addition, a few shear bands and some annealing twins were observed (Figure 2).

Examples of a dislocation microstructure forming inside austenite grains are shown in Figure 2b,c. This is a typical arrangement of highly dense dislocation walls (HDDWs) forming, depending upon austenite grain orientation in one or two slip systems. The distance between the dislocation bands is of the order of 100–200 nm. The formation of the micro-twins of deformation was observed only in a few of the grains, and that is visible as well in a sample after annealing at a temperature of 550 °C for 10 h (Figure 3a,b). In the microstructure, the formation as the result of the spinodal decomposition of carbide κ′ with an L’1_2_-type structure, and the precipitation of the κ-carbide forming along grain boundaries (Figure 3c,d) and twin boundaries (Figure 3a), was observed. κ′-carbides were formed on dislocations inside the grains. The size of the nano-carbides was about 9 nm. Yao et al. [45] found that dislocations moving in the austenitic matrix can shear κ′-carbide precipitation or avoid it, which consequently requires additional energy and leads to the strengthening of the alloy. The process of precipitation along the boundaries occurred as a result of the γ → γ′ + κ reaction, and it became more intensive simultaneously with increases in the aging time. According to the literature [38,46], κ-carbides occur in the form of coarse particles after a long annealing time. A characteristic wave of the concentrations of carbon in the direction of <100> caused, in the areas enriched in carbon, the precipitation of carbide κ′, and the depleted areas still remained austenitic (Figure 4a–c). Carbide κ′ had cube/cube orientation relations ([001]γ‖[001]κ′) towards the austenitic matrix. Contrast is seen in a few areas that is characteristic of spinodal decomposition, probably in its early stages (Figure 4a). The presence of reflexes from the superstructure made it possible to identify those nanoprecipitates with an ordered structure, as shown in Figure 4b,c. Extension in the annealing time up to 100 h at a temperature of 550 °C resulted in the precipitation of carbide κ′ as the result of spinodal decomposition being more advanced (Figure 4d–f). The wavelength was about 15 nm. TEM observations of austenite grains in the specimen after 100 h of aging revealed the presence of spherical nano-sized κ′-carbide particles. Figure 4e shows the dark-field TEM morphologies of samples after 100 h of annealing. An analysis of selected-area diffraction patterns (SAPDs) indicates that the particles among the austenitic matrix are κ′-carbide precipitates. The shape of the κ′-carbides is close to spherical, with a size from 6 to 11 nm. Additionally, the distance between carbides increased from 20 nm up to 30 nm, which is also connected with their growth. For Fe-Mn-Al-C alloys containing 5–10% aluminum, aging at temperatures of 500–650 °C for several hours promotes the growth of ordered nano-sized κ′ carbides [26,36,47].

Chao and Liu [48] found that the composition concentration gradient between the γ-matrix and the κ′-carbide follows a sinusoidal distribution due to the spinodal distribution. The precipitation of κ′-carbides leads to increasing yield strength because κ′-carbides can effectively impede dislocation glide.

Spinodal decomposition generates intragranular nano-sized carbides within the austenite phase [38,39,49]. Various studies have shown variations in alloy characteristics due to different compositions and aging parameters. The formation and location of carbides is the consequence of C and Al content in Fe-Mn-Al-C steels, as proved by Huang et al. [50] and Li et al. [51]. The presence of carbides is observed as intergranular and intragranular precipitations. With the content of Al and C higher than 5.5% and 0.7%, respectively, intergranular precipitations are more likely, but when it is higher than 6.2% and 1.0%, intragranular carbide precipitations are mostly observed. Some works have also reported spinodal decomposition occurring during quenching [32,49,52]. The degree of carbon supersaturation in austenite has been suspected of playing a crucial role in the formation of intragranular carbides during fast cooling [32].

### 3.3. Texture Analysis

The texture of austenite after a 30% deformation was described by the heterogeneous limited fiber α = <110>‖ND, which spread from {110}<001> to {110}<332>. The strongest component in this fiber was the following alloy type {110}<112> orientation: ODF = 3.2.

**Figure 5:**
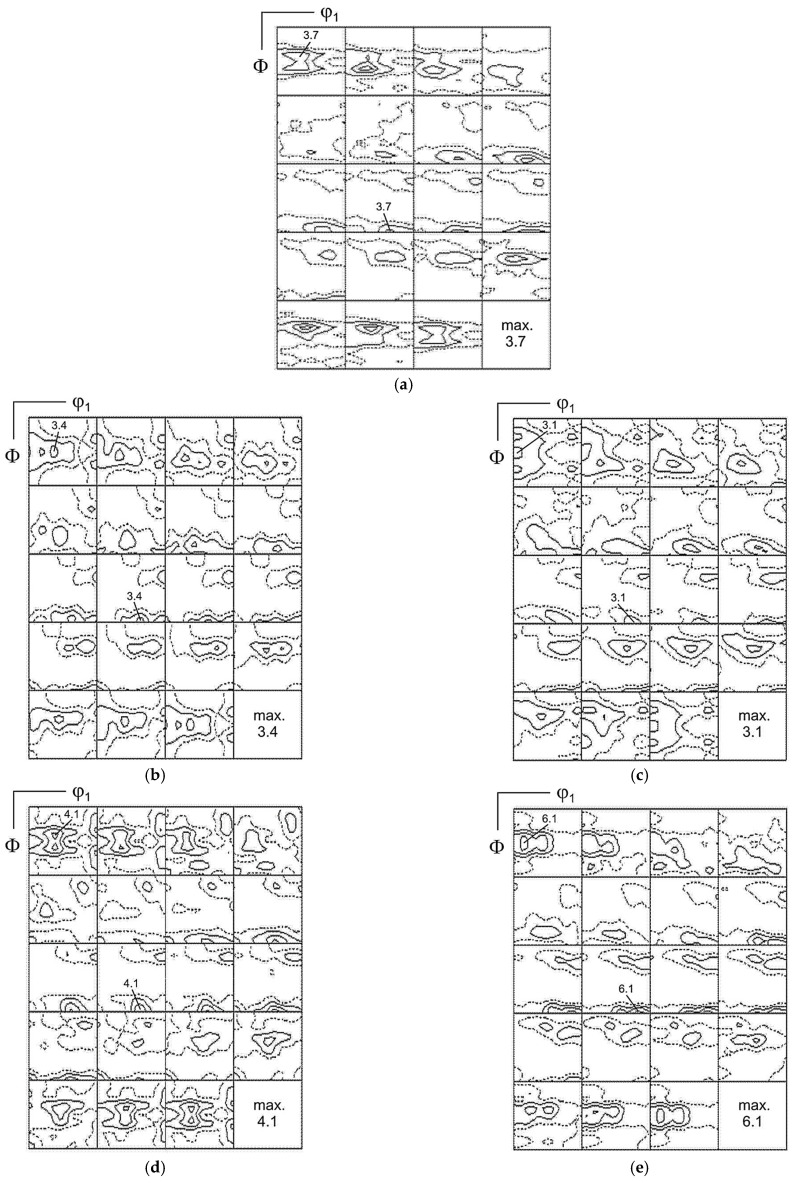
The orientation distribution functions (ODFs) in sections φ_2_ = const for austenite after (**a**) 30% cold rolling and aging at 550 °C for (**b**) 30 min, (**c**) 10 h, (**d**) 24 h and (**e**) 100 h.

**Figure 6:**
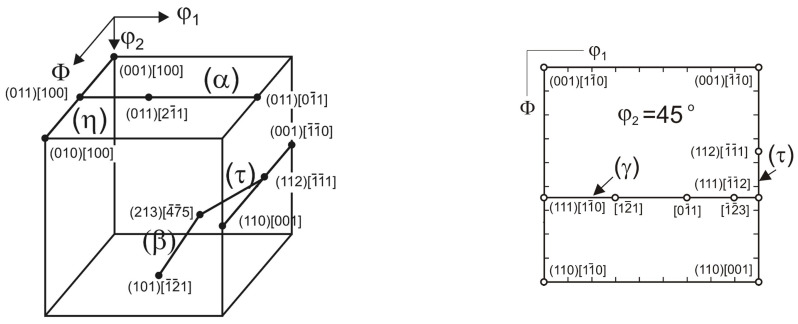
The orientation fibers (α = <110>‖ND, τ = <110>‖TD, β = {110}<112> via {123} <634> to {112} <111>, γ = <111>‖ND, η = <001>‖RD) and ideal orientations that occur in the texture of metals and alloys with FCC structure.

**Figure 7:**
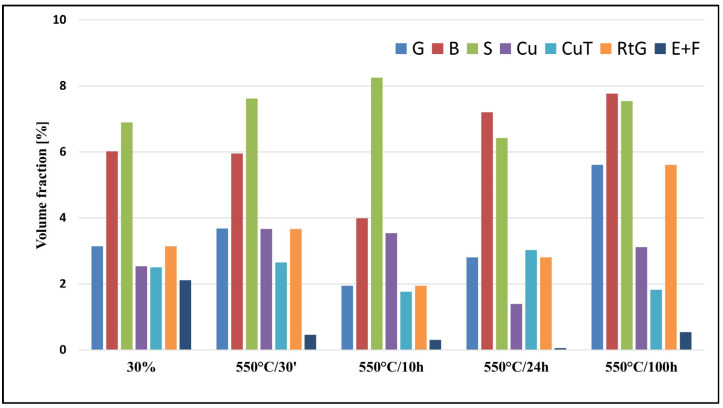
The calculated volume fractions of the important texture components occurring in X85MnAl29-9 steel.

Apart from the α-fiber, limited fibers τ = <110>‖TD and η = <001>‖RD and also single orientations were observed (Figure 5a and Figure 6, Table 3). In the texture after a 30% deformation, the largest volume fraction of texture components was observed for the S-type orientation {123}<634> and the alloy-type component {110}<112> (Figure 7). Similar results were observed in high-manganese steel by Haase et al. [42], Guo et al. [53] and Vercammen [54].

The dominating component of the austenite texture in a sample annealed at a temperature 550 °C for 30 min belonged to the α- and τ-fibers. The maximum corresponded with the alloy orientation {110}<112> (ODFs = 3.4), and a strong copper-type {112}<111> and {113}<332> components were also observed. The strongest orientation of the texture of both fibers was the Goss orientation {110}<001>. In the austenite, however, a new component of the {001}<100> cube orientation locating the η-fiber appeared. As well as that, a cubic orientation {001}<100> representing the η-fiber appeared (Figure 5b, Figure 6 and Figure 7 and Table 3).

After 10 h of aging, the maximum intensity matched the {012}<100> orientation. The {112}<111> and {113}<332> orientations with the τ-fiber were weakened, and so were the orientations situated in the α-fiber, with the exception of the {110}<113> constituent (Figure 5c and Figure 6). In the textures of samples after annealing for 24 and 100 h, an increase in the intensity of the texture of austenite was observed. After 24 h of annealing, the maximum was close to {340}<438> (ODFs = 4.1). The alloy-type {110}<112> and {110}<113> orientations representing (locating along) the α-fiber were strengthened, whereas the copper-type {112}<111> orientation disappeared. In the sample after 100 h of annealing, the maximum intensity matched the {110}<115> orientation. The following orientations were observed as well: Goss {110}<001>, alloy-type {110}<112> and {113}<332>. The α-fiber underwent further strengthening. After annealing at 550 °C for 100 h, an intensification of the degree of austenite texturing was observed (maximum ODFs = 6.1), which can be explained by the processes of precipitation of the carbides. The alloy-type texture was formed during annealing, as usually observed in low-stacking fault energy (SFE) materials (Figure 5, Figure 6 and Figure 7 and Table 3). Only a few works can be found on the texture analysis of TRIPLEX steel for deformed [42,55] and annealed states [56]. Brasche et al. [56] used higher temperatures for the aging process than in the analyzed X85MnAl29-9 steel, and the weakening of the crystallographic texture was explained by oriented nucleation and the resulting annealing twins. The authors found that the carbides formed during annealing did not affect the formed texture. In the tested steel, no significant impact of the precipitation process on the nature of the texture was observed. The increase in the degree of texturing after annealing at longer times above 24 h was caused by the ongoing recovery processes.

### 3.4. Microhardness

**Figure 8:**
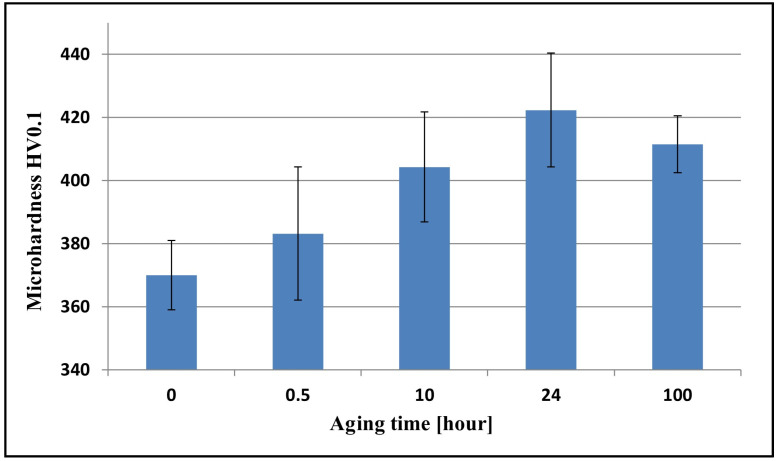
Results of Vickers HV0.1 microhardness measurements after 30% cold rolling and aging at 550 °C for 30 min to 100 h.

Figure 8 presents a microhardness chart depending on the time of aging. The microhardness of the material after a deformation of 30% amounted to 370 HV0.1, and it increased to 422 HV0.1 for a time of aging amounting to 24 h. The mentioned increase in microhardness is most probably due to the presence of a hard-phase κ′-carbide, which is considered the main factor in raising the microhardness in the Fe-Mn-Al-C system [57,58,59].

After annealing for 100 h, the microhardness decreased to the value of 411 HV0.1. This decrease was caused by growth in the precipitations of carbide κ′ in the matrix and κ along the boundaries of the grains of austenite and twins, and alloy aging (Figure 3 and Figure 4). The obtained research results correlate with the work of Moon et al. [60], who stated that the increase in hardness was caused by the precipitation of κ-carbide and, with longer aging times over 50 h, also by the precipitation of β-Mn and ferrite. The decrease in hardness recorded in the tested steel after annealing for 100 h was caused by alloy overaging. A similar tendency in the behavior of the material was observed by Moon et al. [61] in high-manganese steel with the addition of Mo and by Brasche et al. [56] in high-manganese steel.

## 4. Conclusions

As a result of the conducted research, the following conclusions were formulated:

1. The XRD analysis indicated the lattice expansion of austenite and κ′-carbide from the peak shift;
2. The microstructure of X85MnAl29-9 steel after aging at a temperature of 550 °C was composed of an austenitic matrix with carbides κ′ and κ. Small precipitations of carbide κ′ formed in the austenite as a result of spinodal decomposition. The precipitations of carbide κ formed on the boundaries of the austenite grain and along the boundaries of the twins. The process of precipitation along the grain boundaries occurred as a result of the γ → γ′ + κ reaction (austenite depleted in carbon) + κ. An extension in the aging time exerted an influence upon the intensification of spinodal decomposition and resulted in increases in the precipitation of carbides;
3. The process of the precipitation of carbides influenced the development of the austenite texture, principally described by the constituents being parts of the α-fiber: the {110}<001> Goss orientation and the {110}<112> alloy-type orientation;
4. The microhardness of the material increased simultaneously with an extension in the aging time, with the exception of the sample annealed for 100 h. This decrease was the result of the growth in the precipitations of carbide κ′ in the matrix.

Research perspective: The research carried out is an element of a project covering, among others, the application of the heat treatment of high-manganese steels to obtain the optimal mechanical and plastic properties of steel, as well as a quantitative assessment of retained austenite from the perspective of the precipitation processes occurring during artificial aging.

## Figures and Tables

**Table 1:** Chemical composition of X85MnAl29-9 steel under examination, wt.%.

Mn	Al	C	P	S	Fe
29.01	9.52	0.85	0.004	0.012	balance

**Table 2:** Intensity ratios for the pattern (non-textured), material in the initial state (30%) and after aging at 550 °C for various times.

Sample	Iγ_111_/Iγ_200_	Iγ_111_/Iγ_220_
pattern	2.000	3.125
30%	2.977	0.791
550 °C/30′	1.024	0.586
550 °C/10 h	1.181	0.650
550 °C/24 h	0.500	0.749
550 °C/100 h	0.887	0.182

**Table 3:** The texture components and orientation fibers (α, η, τ, β and γ) which occur in the texture of metals and alloys with an FCC structure.

Component	Miller Indices	Euler Angles (φ_1_, ϕ, φ_2_)	Fiber
G (Goss)	(110)<100>	(90°, 90°, 45°)	α, τ,η
B (Brass)	(110)<112>	(55°, 90°, 45°)	α, β
S	(123)<634>	(59°, 37°, 63°)	β
Cu (Copper)	(112)<111>	(90°, 35°, 45°)	τ, β
CuT (Copper Twin)	(552)<115>	(90°, 74°, 45°)	τ
RtG (Rotated Goss)	(110)<110>	(0°, 90°, 45°)	α
E	(111)<110>	(0°, 55°, 45°)	γ
F	(111)<112>	(90°, 55°, 45°)	γ

## Data Availability

The original contributions presented in the study are included in the article, further inquiries can be directed to the corresponding authors.
